# Moyamoya Disease and Syndrome in Caucasian Patients

**DOI:** 10.7759/cureus.37768

**Published:** 2023-04-18

**Authors:** Nuno Neves, Susana Coelho, Natália Marto, Alexandra Bayão Horta, Raquel Gouveia

**Affiliations:** 1 Internal Medicine, Hospital da Luz Lisboa, Lisbon, PRT; 2 Neurology, Hospital da Luz Lisboa, Lisbon, PRT

**Keywords:** revascularization, antiphospholipid antibody, graves’ disease, stroke, moyamoya syndrome, moyamoya disease

## Abstract

Moyamoya disease is a unique cerebrovascular disease characterized by narrowing of the terminal portion of internal carotid arteries and circle of Willis, with consequent development of a network of collateral vessels in response to brain ischemia. Moyamoya vascular pattern can be idiopathic (Moyamoya disease), is more likely to occur in individuals of Asian ascent and in the pediatric age, or is associated with other diseases (Moyamoya syndrome).

We present two cases of stroke in young adults, where workup revealed Moyamoya-type vascular changes. The first case report is of a 42-year-old woman presenting with hemorrhagic stroke, with classic angiographic findings of Moyamoya disease, otherwise asymptomatic. The second case concerns a 36-year-old woman admitted with ischemic stroke; besides the typical angiographic pattern of Moyamoya, the patient was diagnosed with antiphospholipid antibody syndrome and Graves’ disease, two conditions known to be associated with this vasculopathy.

These case reports illustrate the need to consider this entity in the etiological evaluation of ischemic and hemorrhagic cerebrovascular events, even in Western countries, since management and secondary prevention require specific approaches.

## Introduction

Moyamoya disease is a cerebrovascular disease characterized by narrowing of the terminal portion of the internal carotid arteries (ICAs) and/or its main branches in the circle of Willis, with consequent development of abnormal thin collateral vessels mostly at the base of the brain [1].

Although it is idiopathic, there are several underlying clinical conditions that can be associated with this vasculopathy [1,2] such as severe atherosclerosis, cranial radiotherapy, infectious diseases (for example meningitis, especially tuberculosis), hematological diseases (drepanocytosis, β-thalassemia, spherocytosis), autoimmune diseases and vasculitis (Graves’ disease, antiphospholipid antibody syndrome, systemic lupus erythematosus), neurocutaneous syndromes, chromosomal disorders such as Down syndrome, extracranial vascular diseases (renal artery stenosis, coarctation of the aorta, congenital heart diseases and fibromuscular dysplasia), and trauma and tumors affecting the central nervous system. In these cases, the disorder is termed Moyamoya syndrome [3].

Its clinical presentation is variable. Adults present mostly with ischemic events (transient or not) and intracranial hemorrhage, while children present more frequently with brain ischemia [3]. The natural history of Moyamoya disease and syndrome is variable, ranging from a rapid progression with severe disability to a silent course over many years. Bypass between the internal and external carotid circulation, using the superficial temporal and middle cerebral arteries, represents the surgical treatment of choice for ischemic Moyamoya disease [4-6] and, more recently, also for hemorrhagic disease [6-9]. This technique may be combined with indirect revascularization techniques based on synangiosis, in which tissues normally vascularized by branches of the external carotid artery (such as dura mater and temporal muscle) are placed in contact with the ischemic brain surface. In well-selected patients, surgery is the ideal approach to reestablish cerebral hemodynamics and reduce the anomalous collateral network.

We report two cases of stroke in patients with Moyamoya-type vascular alterations, whose presentation, course, and therapeutic approach were challenging.

## Case presentation

Patient A

A 42-year-old Caucasian woman presented to the emergency department four hours after a witnessed syncope, with two minutes duration, from which she recovered noticing weakness in the left arm and leg. She was previously healthy and had no usual medication. Her family history was non-contributory.

**Figure 1:**
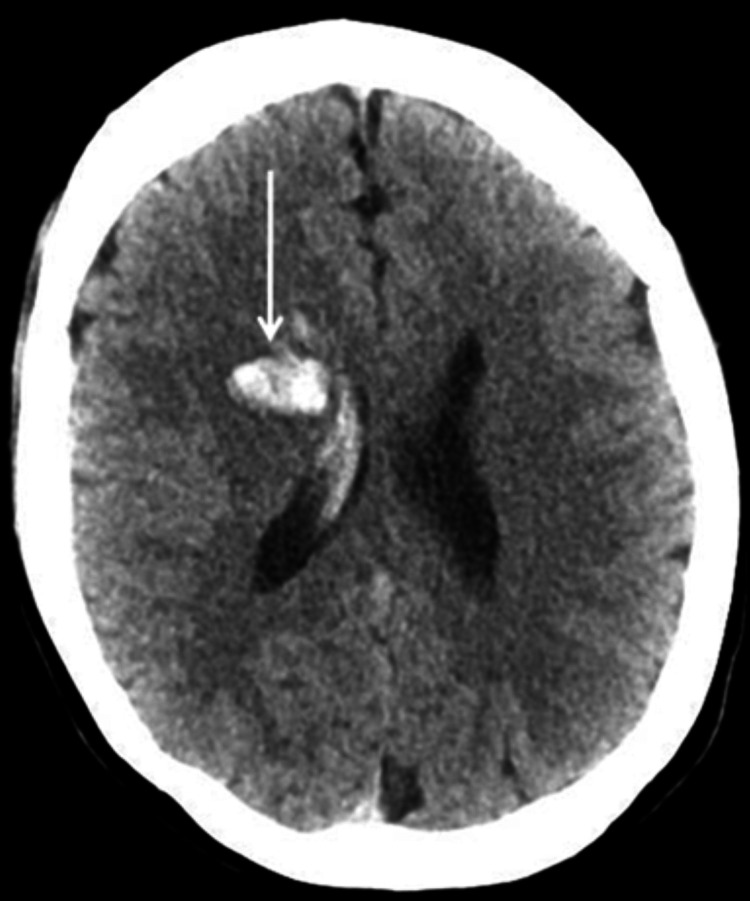
CT showing a right frontal capsular parenchymal hemorrhage (arrow), with rupture into the right lateral and third ventricles CT, computed tomography

**Figure 2:**
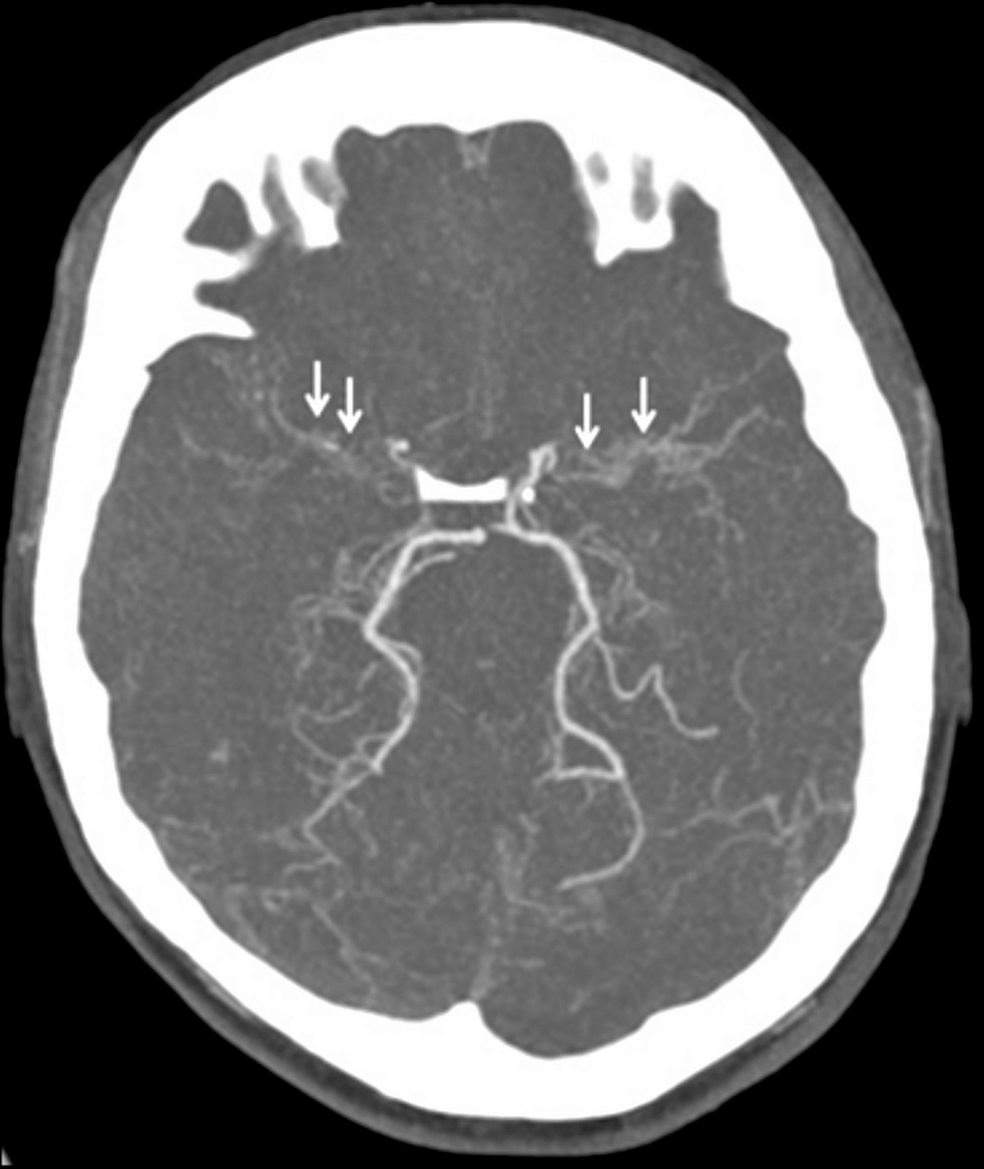
CTA revealing occlusion of both supraclinoid ICAs, with no ACA or MCA individualized bilaterally and a concomitant anomalous vascular network at the base of the brain (arrows) suggestive of Moyamoya vascular pattern (“moyamoya vessels”) CTA, computed tomography angiography; ICA, internal carotid artery; ACA, anterior cerebral artery; MCA, middle cerebral artery

On admission, her blood pressure was 162/90 mmHg, a pulse rate was 80 beats/minute, and oxygen saturation was 99% on ambient air; cardiopulmonary auscultation was unremarkable. Neurological examination revealed left proportionate hemiparesis (muscle strength grade IV/V), with facial involvement, but without sensory or hemispatial deficit, gaze palsy, visual loss, limb ataxia, aphasia, or dysarthria (National Institute of Health Stroke Scale [NIHSS] score of 6). Routine laboratory testing was unremarkable, and brain computed tomography angiography (CTA) revealed an acute right frontal capsular parenchymal hemorrhage, with rupture into the right lateral and third ventricles, determining a millimetric midline shift (Figure 1). Additionally, occlusion of both ICAs was noted in their supraclinoid segment, with a vascular pattern suggestive of Moyamoya disease with multiple dilated lenticulostriate vessels. Anterior cerebral artery (ACA) and middle cerebral artery (MCA) were not visualized bilaterally (Figure 2).

**Figure 3:**
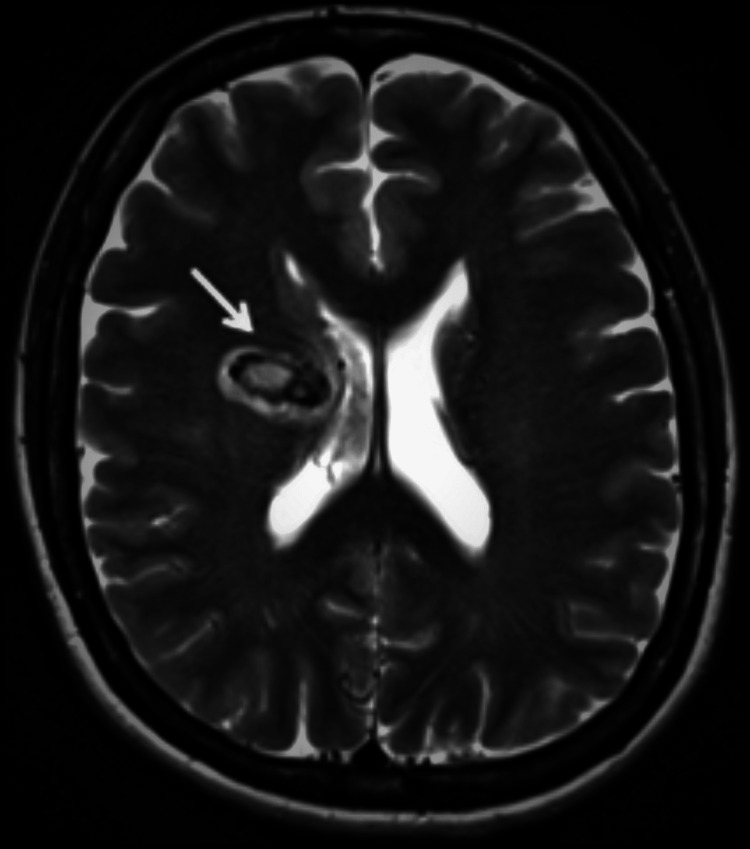
Acute right frontal capsular parenchymal hemorrhage on T2-weighted MRI (arrow) MRI, magnetic resonance imaging

The patient underwent magnetic resonance imaging (MRI) of the brain, which documented a large acute parenchymal hematoma (Figure 3) and multiple small flow voids in the proximal portion of the lateral sulcus.

**Figure 4:**
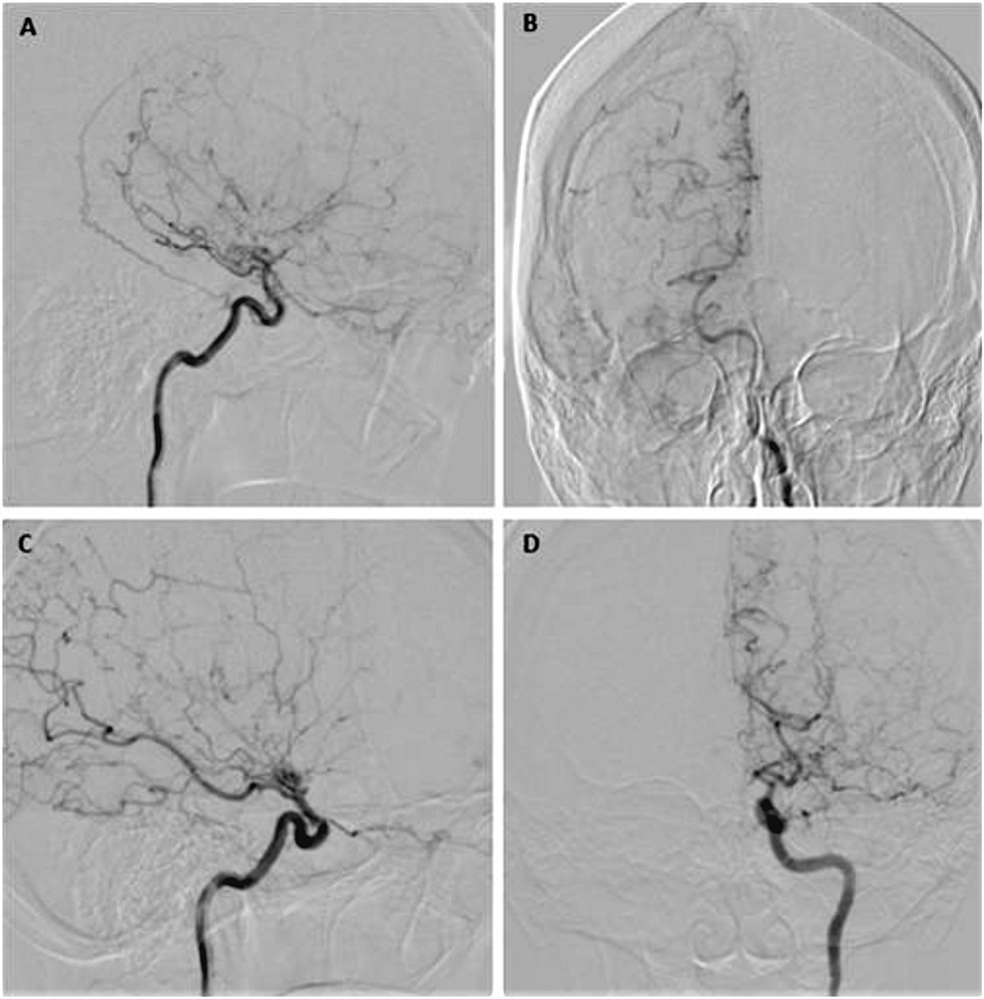
Conventional angiography showing severe occlusion of distal ICA, with no evidence of ACA or MCA as well as slow filling of anomalous perforating arteries (A, B: right side and C, D: left side) ICA, internal carotid artery; ACA, anterior cerebral artery; MCA, middle cerebral artery

Brain angiography confirmed the diagnosis of Moyamoya disease with nearly total distal occlusion of the ICA, absence of ACA and MCA bilaterally, and slow filling of some vicarious perforating arteries (Figure 4; panel A and B: right side; panel C and D: left side). Posterior cerebral arteries (PCA) and infratentorial circulation had normal morphology, with distal leptomeningeal compensatory vicarious circulation, as well as the proximal perforating branches.

The remaining stroke workup was unremarkable, including 24-hour Holter monitoring, transthoracic echocardiogram, and the screening for metabolic alterations that contribute to atherosclerosis, such as diabetes mellitus and familial dyslipidemias. The etiological study directed to the clinical conditions associated with this vasculopathy was negative, in particular hematological diseases, autoimmune diseases such as Graves’ disease and Hashimoto’s thyroiditis, vasculitis, antiphospholipid antibody syndrome, and systemic lupus erythematosus.

At the time of discharge, she had recovered full strength in her limbs, yet maintaining a minor left facial paresis. She was proposed to undergo bilateral elective revascularization surgery, which was uneventful. No new vascular events were observed thereafter, and, at three years of follow-up, no sequelae were evident.

Patient B

A 36-year-old Caucasian woman presented to the emergency department with sudden onset of right leg numbness and weakness, which had started 20 hours prior to admission. The patient had a previous history of hypertension and obesity, usually under medication with lercanidipine, irbesartan/hydrochlorothiazide, and nebivolol. In the six months prior to hospitalization, there was a description of progressive and unexplained behavioral disturbance, which was characterized by a compulsion for games and gambling, running into debt, and subsequent job loss. Neither the patient nor her family sought professional help for the notorious behavioral changes.

**Figure 5:**
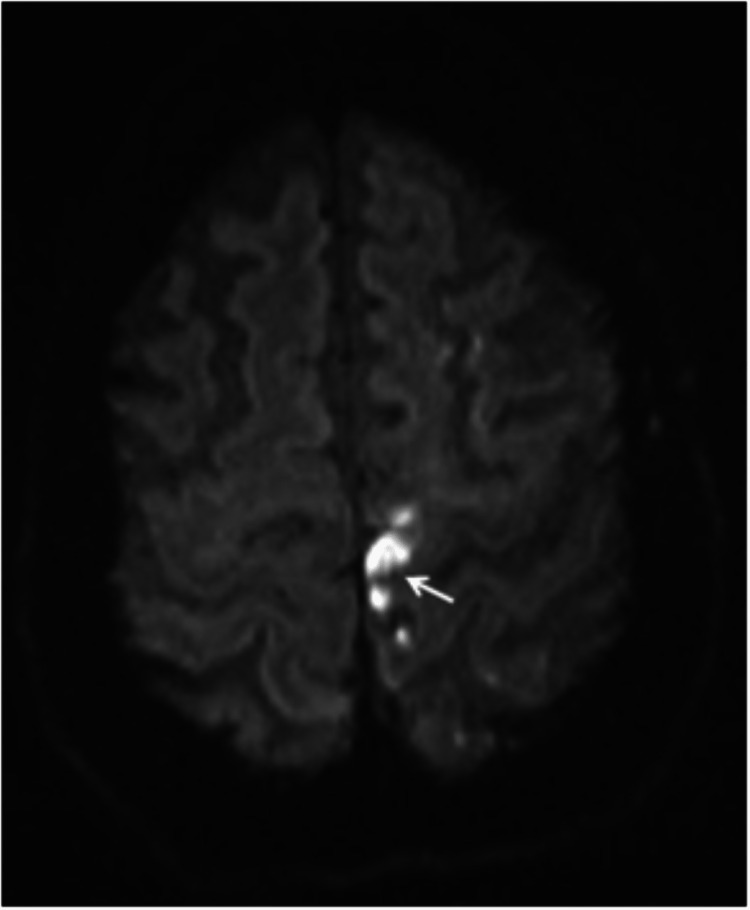
Diffusion-weighted MRI showing a left internal fronto-parietal cortical ischemic lesion (arrow) MRI, magnetic resonance imaging

On admission, her blood pressure was 173/98 mmHg, pulse rate was 63 beats/minute, and oxygen saturation was 99% on ambient air. The observation was unremarkable except for right lower limb monoparesis with hypoesthesia (NIHSS 3) and exophthalmia. Brain computed tomography (CT) identified multiple ischemic sequelar lesions, and brain MRI documented an acute left internal fronto-parietal cortical ischemic lesion (Figure 5), frontal lobe atrophy, and multiple chronic ischemic lesions.

**Figure 6:**
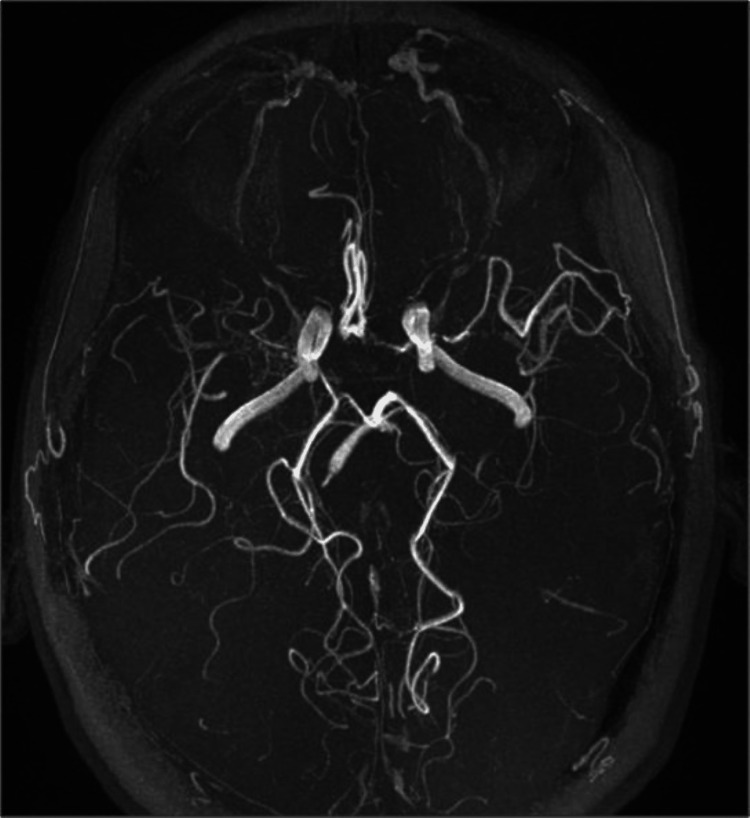
TOF-MRA demonstrating terminal internal carotid artery stenosis and abnormal vascular network formation bilaterally; simultaneously, there is near occlusive stenosis of right M1 segment and stenosis of the left A1 and M1 segments, with indistinguishable right A1 segment, suggesting Moyamoya vasculopathy TOF-MRA, time-of-flight magnetic resonance angiography

Magnetic resonance angiography (MRA) study noted bilateral stenosis of the supraclinoid segment of the ICA, with a near occlusive stenosis of the right M1 segment and stenosis of the left A1 and M1 segments. The right A1 segment was very thin, and there was significant hypertrophy of the lenticulostriate branches, suggesting Moyamoya vasculopathy (Figure 6).

**Figure 7:**
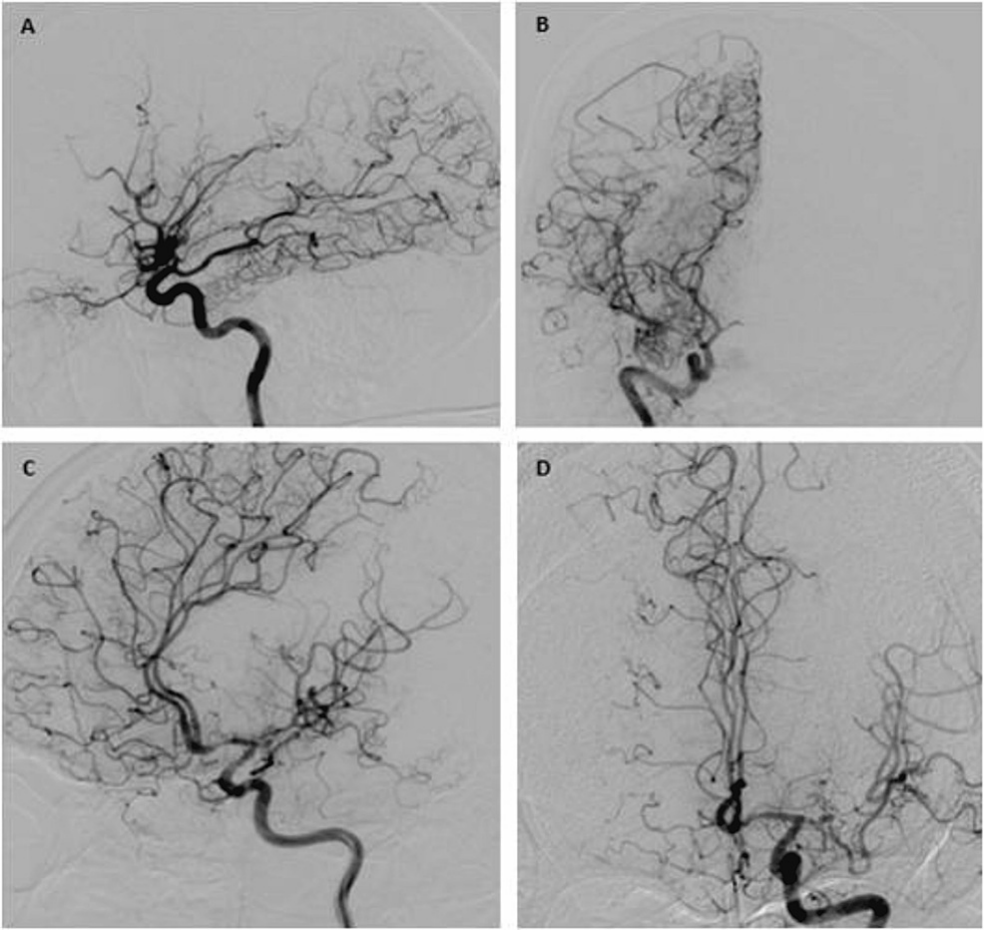
Conventional angiography showing supraclinoid stenosis of both ICAs, left A1, and M1 segments bilaterally, while right A1 is not visualized (A, B: right side and C, D: left side) ICA, internal carotid artery

On perfusion study, it was possible to individualize some areas of reduction in cerebral blood flow (CBF), namely in right fronto-parietal transition (border territory), right frontal and parietal posterior topography (distal cortical territory of right MCA), and middle and upper left frontal region (territory of left MCA), with the latest also with reduction in cerebral blood volume (CBV), in the context of ischemia. CBV images showed no reduction in the right parietal and right parieto-occipital junctional areas, suggesting that, through a leptomeningeal anastomotic pattern, this circulation was balanced. The angiography revealed multiple focal stenoses in the supraclinoid portion of both ICAs, M1 segments bilaterally, and left A1, while right A1 segment was not visualized (Figure 7; panel A and B: right side; panel C and D: left side). There were leptomeningeal anastomoses between right PCA and homolateral MCA and between ACA and MCA bilaterally; the anastomoses between internal and external lenticulostriate arteries allowed blood supply of M1 segments; no vertebrobasilar stenoses were noted.

The remaining study revealed aortic bicuspidia on transesophageal echocardiography, with no signs of potentially embolic heart disease. Carotid and vertebral Doppler ultrasound showed no significant changes. CTA of the body showed no extracranial vascular involvement.

Laboratory workup was remarkable for positive antiphospholipid antibodies, which remain positive at 12 weeks’ re-evaluation, confirming the diagnosis of antiphospholipid antibody syndrome (Table 1). Additionally, euthyroid Graves’ disease was diagnosed with increased thyroid antibodies (Table 1). Diagnostic workup of other conditions known to be related to Moyamoya vasculopathy, such as hematological diseases, vasculitis, and other autoimmune diseases, such as systemic lupus erythematosus or connective tissue diseases, was negative.

**Table 1:** Laboratory workup of patient B confirming antiphospholipid antibody syndrome and Graves' disease U/mL, units per milliliter; IU/L, international units per liter; IU/mL, international units per milliliter Ig, immunoglobulin; TRAb, thyroid stimulating hormone receptor antibody; TPO-Ab, thyroid peroxidase antibody; Tg-Ab, thyroglobulin antibody

Antibodies	Week 0	Week 12	Reference value
Lupus anticoagulant	Positive	Positive	-
IgM anticardiolipin antibodies (U/mL)	46	41	<15
IgG anticardiolipin antibodies (U/mL)	145	137	<30
IgM anti-β2 glycoprotein I antibodies (U/mL)	12	14	<7
IgG anti-β2 glycoprotein I antibodies (U/mL)	7,9	8	<7
TRAb (IU/L)	4,8	-	<1.5
TPO-Ab (IU/L)	1145	-	<60
Tg-Ab (IU/mL)	1158	-	<60

She was started on anticoagulation with warfarin and was not considered candidate for revascularization surgery due to subacute behavioral disturbance resulting from frontal atrophy, permanent neurological deficit, and high surgical risk imposed by her associated diseases, namely antiphospholipid antibody syndrome.

**Figure 8:**
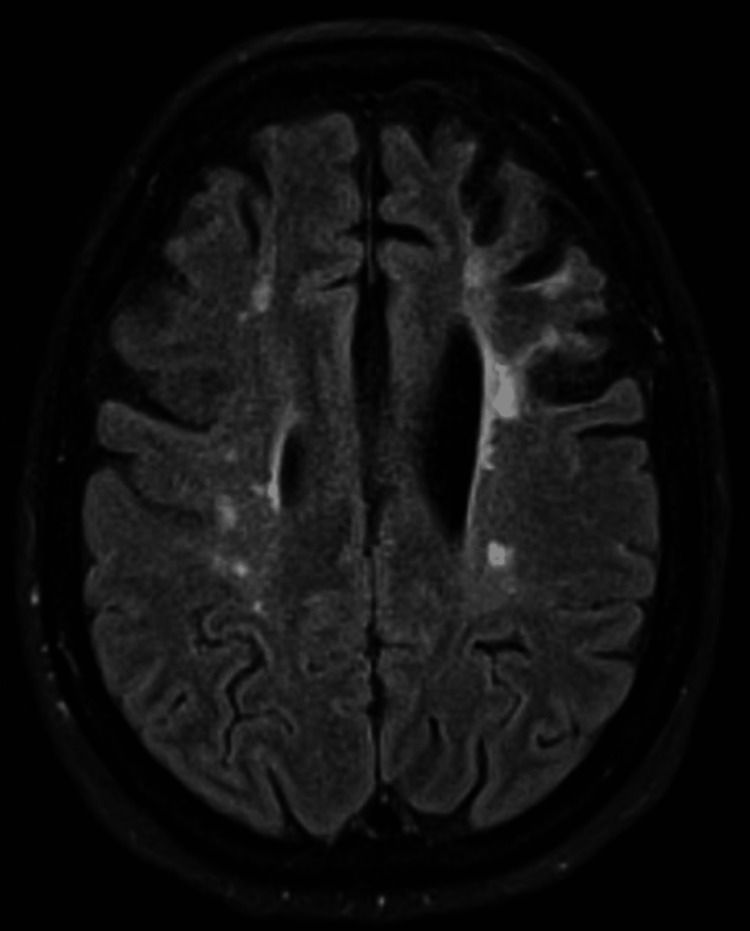
FLAIR-weighted MRI showing a corticosubcortical frontal hyper-intensity with, mostly, frontal cerebral atrophy FLAIR, fluid-attenuated inversion recovery; MRI, magnetic resonance imaging

**Figure 9:**
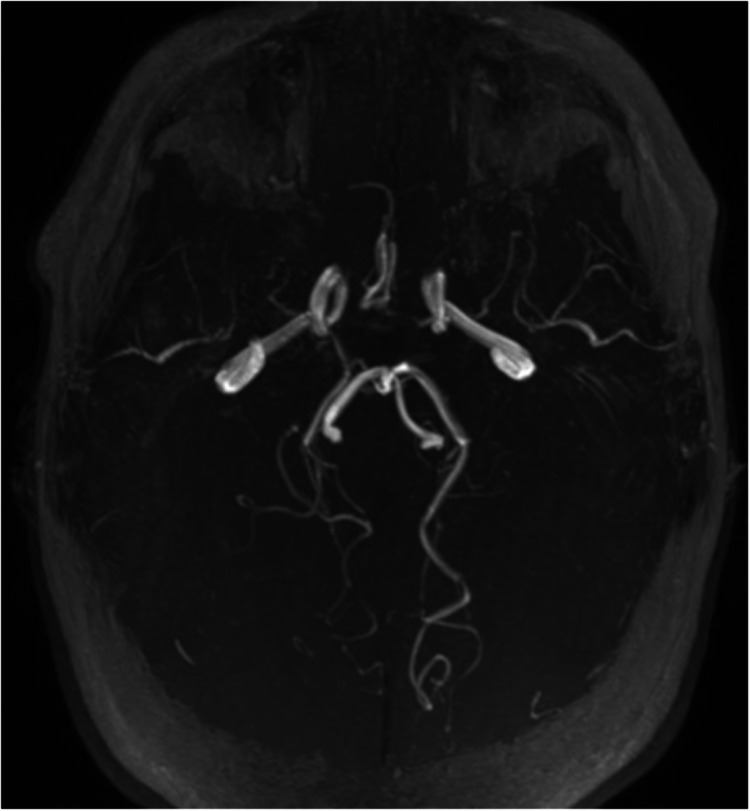
TOF-MRA demonstrating bilateral stenosis of the supraclinoid segment of the ICA, with a near occlusive stenosis of the right M1 segment and stenosis of the left A1 and M1 segments, while right A1 segment was not visualized (same findings of initial angiography) TOF-MRA, time-of-flight magnetic resonance angiography; ICA, internal carotid artery

Over the next three years, there was persistent cognitive decline with neuropsychological tests revealing impaired executive and judgment capacity, maintaining a discrete right lower limb paresis and apraxia. As a consequence of chronic hypoperfusion, previously demonstrated by the reduction in CBF and, occasionally, CBV, follow-up MRI showed an important frontal atrophy with cortical predominance, qualitatively exacerbated compared to the previous MRI (Figure 8). Similarly to previous imaging, MRA documented bilateral stenosis of the supraclinoid segment of the ICA, with a near occlusive stenosis of the right M1 segment and stenosis of the left A1 and M1 segments, while right A1 segment was not visualized (Figure 9).

## Discussion

Moyamoya is a rare and progressive cerebrovascular disease characterized by narrowing of the terminal (supraclinoid) portion of the ICA and/or its main branches, with subsequent development of small and anomalous collateral vessels at the base of the brain. These vessels have a typical smoky appearance on angiography, first called “moyamoya,” a Japanese expression meaning hazy like a “puff of smoke” [3]. In this disease, there is a unique tendency for cerebral irrigation to be diverted from the circulation by the internal carotid to the external carotid system [9]. Insufficiency of this system can lead not only to cerebral ischemia but also to hemorrhage by the inadequate anastomoses formed [9].

Moyamoya disease is rare in western countries, and its occurrence is around 10 times higher in East Asian countries, with the highest prevalence in Japan [3]. Its onset has a first peak in childhood between 5 and 10 years and a second peak in the fourth decade of life [3]. In Japan, in 10-15% of the cases, there is a positive family history [3]. In the East Asian population, a gene - *RNF213* - located on chromosome 17 (17q25-ter) has been implicated as a susceptibility factor for the disease/syndrome [10,11]. However, its exact function is yet to be determined [10]. A polymorphism in its structure was identified in 95% of the cases with family history and in 79% of sporadic cases, correlating with an earlier onset of symptoms and more severe forms of the disease [10,11], making it a good prognostic marker.

As a disease, Moyamoya is idiopathic, but there are several clinical conditions associated with this vasculopathy. Hayashi et al. [12], in a study in Japan, reported atherosclerosis as the disease most often associated with Moyamoya syndrome (29%), followed by Down syndrome (15.1%), Recklinghausen disease (14%), and, finally, brain tumors (7.5%). In Taiwan, atherosclerosis (32.4%) is immediately followed by thyroid disease (29.7%) as associations with Moyamoya [13]. Multiple inflammatory pathways in the above-mentioned diseases may promote the onset or progression of the syndrome [1].

Our second case is of particular interest, since Moyamoya is a rare vasculopathy (mainly in western countries), especially in association with two autoimmune diseases (Graves’ disease and antiphospholipid antibody syndrome). In Graves’ disease, elevated thyroid hormone levels are believed to lead to progression of Moyamoya syndrome by increasing vascular sensitivity to the sympathetic nervous system. However, even euthyroid patients with high levels of anti-thyroid antibodies (namely TRAb and TPO-Ab) may have stenosis in the terminal portion of the ICA [14], much like patient B. Although Graves’ disease affects 0.5% of the population, its coexistence with Moyamoya syndrome is rare and seen, mostly, in East Asian countries [2], with less than one hundred cases in which a Moyamoya vasculopathy associated with Graves’ disease has been described [15]. Association with Hashimoto's thyroiditis is more common in patients in western countries than Graves' disease and has been reported as a risk factor for a worse outcome after revascularization surgery [2], emphasizing the importance of excluding thyroid disease and assure its proper control before considering surgery. Although antiphospholipid antibody syndrome is recognized as a Moyamoya vasculopathy related condition, the casual relationship between both is not clear. On the one hand, alterations in endothelial cells in Moyamoya vasculopathy can further predispose to formation of antiphospholipid antibodies, which can react with the phospholipid component of endothelial cell membranes perpetuating vascular damage; on the other hand, some researchers have speculated that the development of a Moyamoya vascular pattern in those with antiphospholipid antibody syndrome is secondary to thrombosis and stenosis in basal cerebral vasculature [16]. In 2014, Wang et al. carried out a literature review in which there was a mention of 16 cases of Moyamoya syndrome in association with antiphospholipid antibodies, with three of them also with autoimmune thyroid disease. From these sixteen patients, only five had criteria for antiphospholipid antibody syndrome [17], corroborating the scarcity of well documented cases of concomitant Moyamoya syndrome and antiphospholipid antibody syndrome in literature, as well as the importance of excluding other associated conditions.

The clinical presentation of Moyamoya disease and syndrome is variable, as illustrated by the cases herein presented. In fact, our patients had opposite presentations: patient A presented with an acute focal neurological deficit caused by an intracerebral hemorrhage, from which she had total recovery, while patient B presented with an acute focal neurological deficit of ischemic etiology, superimposed on a behavioral disorder with a subacute course, subsequent cognitive decline, and residual neurological deficit. Regarding the clinical presentation, adults can present with either ischemic or hemorrhagic stroke, while children have more frequently ischemic events [3], which can be triggered by events that induce cerebral vasoconstriction in response to carbon dioxide decrease. More than 20% of adults with Moyamoya disease present with intracranial hemorrhage, yet in western countries this presentation appears to be more rare than in Asia [3].The existence of choroidal and/or thalamic collaterals, PCA involvement, and a higher Suzuki stage (Table 2) increase the risk of bleeding as presentation [9]. Hemorrhage occurs either due to rupture of deep fragile collaterals, leading to intraparenchymal/intraventricular hemorrhage, or rupture of saccular aneurysms in the Willis circle (subarachnoid hemorrhage), formed in response to shifting circulatory patterns [3].

**Table 2:** Staging system of Moyamoya disease Based on a proposal by Suzuki et al. [2,3,18] ICA, internal carotid artery; ACA, anterior cerebral artery; MCA, middle cerebral artery

Grades	Description
I	Narrowing of carotid fork
II	Initial appearance of moyamoya vessels, with stenosis of terminal branches of ICA (ACA and/or MCA)
III	Intensification of moyamoya vessels, with deep vessels showing a "puff of smoke" appearance
IV	Minimization of moyamoya vessels, since deep moyamoya vessels begin to regress while transdural collaterals begin to appear
V	Reduction of moyamoya vessels with progression of transdural collateral vessels
VI	Disappearance of moyamoya vessels, total occlusion of ICA with blood supply mainly from the external carotid artery

Adding to cerebrovascular complications, cognitive impairment can occur even in patients who have never experienced an ischemic event, as a consequence of the deleterious effects of chronic hypoxemia. Certainly, this was what happened to patient B even before the first clinically evident stroke. Vascular epilepsy due to cortical ischemia may also occur as well as movement disorders, secondary to vascular damage to the basal ganglia. Headache and migraine, frequently, associated with this pathology can, possibly, be explained by the stimulation of dural nociceptive receptors by dilated transdural collateral vessels [3].

The natural history of Moyamoya disease and syndrome is unpredictable and may evolve rapidly with disability and neurological decline or remain silent over many years. In adults, the progression appears to be milder with progressive loss of autonomy in 20% of patients (60% in children) [3]. In symptomatic adults, the rate of ischemic accidents is around 10-15% per year, and in asymptomatic, it is 3% per year [3]. If revascularization surgery is performed before neurological damage is established, the prognosis tends to be excellent, even in patients with severe angiographic findings [19]. Despite an important hemorrhage as presentation in patient A, her recovery was almost complete and the surgical procedure uneventful, with no sequels thereafter. Contrasting patient B presented with progressive decline and was not considered a candidate for surgery due to neuropsychiatric deterioration and permanent neurological deficit. The evidence on MRI of frontal lobe atrophy implies a worse prognosis, and, despite the best medical treatment, neither regression of the deficits nor definitive resolution of her vasculopathy is expected.

Both of our patients had typical bilateral involvement as required by previous diagnostic criteria for the disease/syndrome. However, the increased number of patients with evidence of unilateral involvement [9-13] and the recognition that such cases can progress to bilateral involvement [9] motivated a revision of the diagnostic criteria in 2015. Accordingly, in patients with unilateral involvement or atherosclerosis, cerebral angiography is required to confirm the diagnosis, while patients with bilateral involvement may be diagnosed either by angiography or MRA [6]. With this reviewed criteria, patients with unilateral findings have Moyamoya disease/syndrome confirmed since cerebral angiography shows typical features.

Internal and external carotid circulation bypass, using the superficial temporal and middle cerebral artery, has been one of the strategies used to minimize the ischemia and hemorrhage associated with this disorder. Currently, it represents the first-line treatment of ischemic Moyamoya disease [4-6]. For those with predominantly hemorrhagic manifestations, the best therapeutic approach is still controversial, although recent evidence suggests that the same surgical approach may decrease the hemorrhagic recurrence [6-9]. Accordingly, this constituted our therapeutic choice in patient A. Regarding procedural complications, perioperative cerebral ischemia and cerebral hyperperfusion syndrome are the main risks. Cerebral ischemia can be caused by, at least, three mechanisms: “watershed shift phenomenon”, thromboembolism at the anastomosis site, and mechanical compression of the brain surface by the swollen temporal muscle used for indirect bypass [9]. Watershed shift is a characteristic pathophysiological phenomenon consisting of ischemia of the cortex nearby the bypass. In these cases, retrograde blood supply from the bypass, between the superficial temporal artery and MCA, may interfere with the anterograde flow from proximal MCA and, thus, lead to a temporary decrease in blood flow at the cortex supplied by the adjacent branch of MCA [9]. On the other hand, the rapid increase in blood flow at the anastomosis site may lead to focal hyperemia associated with vasogenic edema and/or hemorrhagic transformation, which is one of the most serious complications of revascularization surgery in Moyamoya disease and syndrome [6,9,20]. The prognosis of focal neurological signs associated with hyperperfusion syndrome is generally good but may, rarely, lead to late intracerebral and/or subarachnoid hemorrhage [9,20]. The incidence of symptomatic late bleeding due to hyperperfusion is 3.3% [20]. In order to prevent it, proper blood pressure control is essential, while avoiding hypotension as this may lead to secondary ischemia. There are also indirect revascularization techniques based on synangiosis, in which tissues normally vascularized by branches of the external carotid artery (such as dura mater and temporal muscle) are placed in contact with the ischemic brain surface [3]. Neovascularization from these tissues is thought to be achieved by the release of angiogenic factors by the ischemic brain tissue, taking about three months for an efficient vascular network to develop. There is no formal recommendation for one procedure over another. However, indirect revascularization has been preferred in children because of the technical difficulty of the bypass using small vessels, whereas in adults direct revascularization has been favored [3].

Medical management includes antiplatelet therapy in all patients with ischemic manifestations and may be the only strategy in those who lack favorable surgical conditions or in patients whose revascularization surgery is postponed by recent vascular events. Dual antiplatelet aggregation is not recommended because of the associated increased intracerebral hemorrhagic risk [3]. In certain conditions such as antiphospholipid syndrome, anticoagulation is recommended like in the general population, as well as the appropriate treatment of any underlying pathologies. Patients with Moyamoya disease or syndrome may have hypertension, which should be treated in order to avoid future ischemic and hemorrhagic events, having in mind that hypotension and hypovolemia may precipitate additional ischemic events. Other cardiovascular risk factors such as dyslipidemia, obesity, and diabetes mellitus should be aggressively managed with lifestyle modification and recommended medication. Simultaneously, sedentary lifestyle and smoking are strongly discouraged due to the increased risk of cerebrovascular complications presented by these patients.

Regarding surveillance and prevention, in those with asymptomatic imaging alterations compatible with Moyamoya vasculopathy, aspirin is recommended as long as imaging surveillance by transcranial Doppler, MRA, or CTA (with or without perfusion studies to evaluate hemodynamics) is ensured. In these patients, surgery is recommended if signs or symptoms attributable to this vasculopathy appear or if imaging studies show compromised cerebral perfusion. In those with ischemic and/or hemorrhagic manifestations, referral for revascularization surgery is indicated if there is no established neurological deficit.

## Conclusions

In young adults with sudden onset of focal neurological deficits, it is important to suspect of less frequent causes of stroke, including Moyamoya disease and syndrome, even in Western countries, as this disease has specific therapeutic and prognostic implications. Once Moyamoya-type vascular alterations are found, efforts should be made to exclude concomitant conditions associated with this vasculopathy so that targeted treatment can be instituted. Despite the natural course of the disease and because the syndrome is variable and unpredictable, revascularization surgery is known to have good results, even in cases with severe angiographic findings, if performed before the onset of definitive neurological damage.
